# A Novel User Emotional Interaction Design Model Using Long and Short-Term Memory Networks and Deep Learning

**DOI:** 10.3389/fpsyg.2021.674853

**Published:** 2021-04-20

**Authors:** Xiang Chen, Rubing Huang, Xin Li, Lei Xiao, Ming Zhou, Linghao Zhang

**Affiliations:** ^1^School of Design, Jiangnan University, Wuxi, China; ^2^Faculty of Information Technology, Macau University of Science and Technology, Macau, China

**Keywords:** interaction design, emotion recognition, LSTM, speech, self-attention mechanism

## Abstract

Emotional design is an important development trend of interaction design. Emotional design in products plays a key role in enhancing user experience and inducing user emotional resonance. In recent years, based on the user's emotional experience, the design concept of strengthening product emotional design has become a new direction for most designers to improve their design thinking. In the emotional interaction design, the machine needs to capture the user's key information in real time, recognize the user's emotional state, and use a variety of clues to finally determine the appropriate user model. Based on this background, this research uses a deep learning mechanism for more accurate and effective emotion recognition, thereby optimizing the design of the interactive system and improving the user experience. First of all, this research discusses how to use user characteristics such as speech, facial expression, video, heartbeat, etc., to make machines more accurately recognize human emotions. Through the analysis of various characteristics, the speech is selected as the experimental material. Second, a speech-based emotion recognition method is proposed. The mel-Frequency cepstral coefficient (MFCC) of the speech signal is used as the input of the improved long and short-term memory network (ILSTM). To ensure the integrity of the information and the accuracy of the output at the next moment, ILSTM makes peephole connections in the forget gate and input gate of LSTM, and adds the unit state as input data to the threshold layer. The emotional features obtained by ILSTM are input into the attention layer, and the self-attention mechanism is used to calculate the weight of each frame of speech signal. The speech features with higher weights are used to distinguish different emotions and complete the emotion recognition of the speech signal. Experiments on the EMO-DB and CASIA datasets verify the effectiveness of the model for emotion recognition. Finally, the feasibility of emotional interaction system design is discussed.

## Introduction

**Figure 1:**
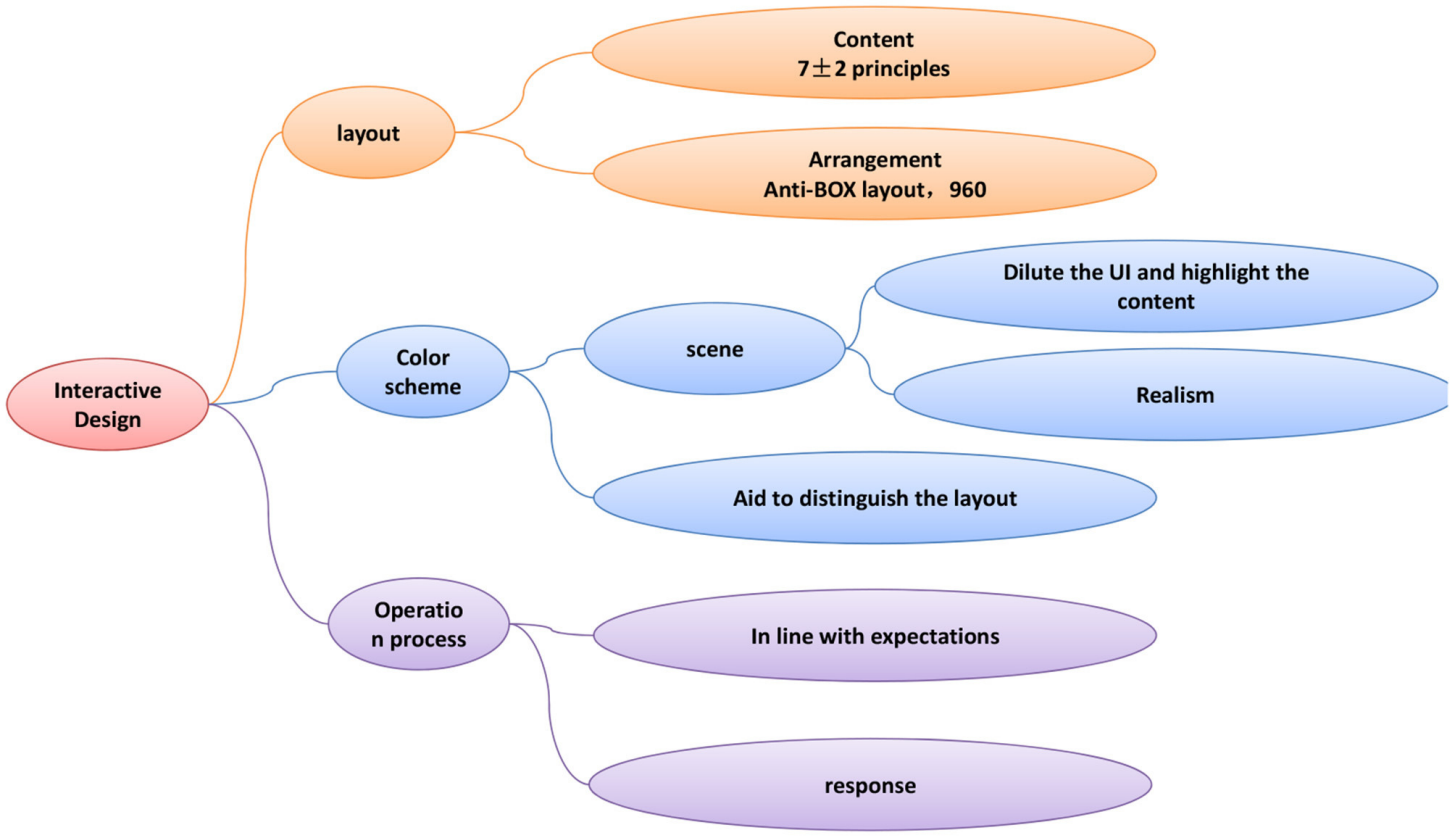
The relationship between user experience and interaction design.

In the 19th century, some designers in Europe proposed that the fundamental purpose of design is to serve people. Design should be people-oriented and emphasize the importance of people in the composition of design. If the design only has a good appearance, but does not have practicality and functionality, then the design is not a qualified work. Especially in today's society, people have begun to pursue spiritual pursuits. Designers must actively focus on user experience and emphasize user experience functions in design. Only in this way, design is what society needs and recognizes. Therefore, user experience has become an extremely important element, and it is the key that any designer should pay attention to. The relationship between user experience and interaction design is shown in Figure 1.

Good interaction design focuses on user experience. A good user experience is able to adapt to different types of users and the different emotions of each user. Therefore, emotion recognition is the key to good interaction design. The accuracy of emotion recognition is the top priority of interaction design. Emotion recognition is the process of using computers to automatically recognize human emotional states (Shu et al., 2018; Marco-Garcia et al., 2019; Ahmed et al., 2020; Assed et al., 2020; Nyquist and Luebbe, 2020). Due to the richness of human emotion expression, emotion recognition can use different combinations of multiple emotion expression materials to make judgments. In the historical process of emotion recognition research, scholars have researched from single-modal modeling (Edgar et al., 2020; Gosztolya, 2020; Panda et al., 2020), hybrid multi-modal modeling (Ayata et al., 2020; Zhang et al., 2020), to complex deep neural networks (Abdulsalam et al., 2019; Aouani and Ayed, 2020; Sharma et al., 2020). The relationship between human emotion and its multiple expressions is gradually being unearthed. There are many ways for humans to express emotions, and the carriers of emotions are also very rich, but the most intuitive among the many emotional carriers are language and expressions. If a person tends to use language to express their emotions, then the person's speech data may contain more emotion discrimination information. If a person tends to use facial expressions to express their emotions, then the person's video data may carry more emotion discrimination information.

The current emotion recognition is mainly developed from the following aspects. One is the different types of experimental data. Various forms of user physiological and behavioral data will be generated during the interaction. A variety of sensors are usually used in detecting emotional features, and the physiological state or behavior data of the user is captured without interpreting the input data. For example, a camera can capture facial expressions, body postures or gestures, a microphone can capture speech information, and a physiological sensor can directly measure physiological values such as skin temperature and heartbeat. Current popular researches mainly focus on physiological signals (Shu et al., 2018; Ayata et al., 2020), facial expressions (Martinez-Sanchez et al., 2017; Dey et al., 2019; Sivasangari et al., 2019), body postures (Bijlstra et al., 2019; Lenzoni et al., 2020) and speech information (Issa et al., 2020; Parthasarathy and Busso, 2020; Wang et al., 2020; Zhang et al., 2021). For different types of data, different types of features are extracted. Visual features are commonly used in sentiment analysis. Many studies have shown that the generation of human emotions is inseparable from what people see. For example, people often feel fear in a dark environment, and they will feel comfortable to see the symmetrical rules. Visual features include color features, texture features, SIFT features (Jayasimha and Reddy, 2019), HOG features (Bharate et al., 2019) and so on. Audio features include MFCC features (Sheikhan et al., 2012), Audio-Six features (Krishna et al., 2019). Attribute characteristics include Classemes characteristics (Torresani et al., 2010), Senti Bank characteristics (Borth et al., 2013). Second, the method of feature extraction is different. As emotion recognition uses different data types, the feature extraction methods for different types of data will also be different. Commonly used feature extraction methods are CNN (Brousseau et al., 2020; Cabada et al., 2020), LSTM (Wu et al., 2019; Zou et al., 2020), PCA (Smallman et al., 2020), etc. The third is the difference in classification models. Commonly used classification models are Support Vector Machine (SVM; Vapnik, 1999), Random Forest (RF; Svetnik et al., 2003), Hidden Markov Model (HMM; Lay et al., 2003), Gaussian Mixture Model (GMM; Navyasri et al., 2018), Artifical Neural Network (ANN; Powroznik, 2014) and the Soft-max regression classifier widely used in deep learning (Liu and Zheng, 2007).

Among the many researches on emotion recognition, the research on emotion recognition based on speech is the most popular. In speech-based emotion recognition, deep learning can be used not only for classification, but also for feature extraction. Deep learning has excellent performance in extracting discriminative speech emotional features, especially in the extraction of high-level emotional feature representations of large samples. Literature (Jiang et al., 2019) proposed a speech emotion recognition system based on recurrent neural network (RNN). The system uses a powerful learning method with bidirectional long and short-term memory (BLSTM) to extract high-level representations of emotional states from the time dynamics of emotional states, which improves recognition accuracy. Literature (Trigeorgis et al., 2016) proposed an end-to-end method that directly uses speech signals as input. Through the combination of convolutional neural network (CNN) and LSTM network, “context awareness” is realized. The performance of this method on the RECOLA database is better than traditional methods based on signal processing technology to extract features. Literature (Sak et al., 2014) uses LSTM for speech emotion recognition, LSTM converges fast and the model is small. Multiple units in the LSTM network form a tandem structure. The LSTM is created to avoid the long-term dependence of RNN affected by the state of a long time ago. In the three gating units, the calculation of each unit will lose the information processed in the previous sequence, and it is difficult to ensure the integrity of the information and the accuracy of the output at the next moment. Due to the low accuracy of the above methods in speech emotion recognition and the lack of information in the extracted speech signal features, this study uses a speech emotion recognition method based on LSTM and self-attention mechanism. This method uses MFCC as the emotional feature, uses LSTM to learn the temporal relevance of the speech sequence, and calculates the weight of each frame of speech signal in the emotional feature through the attention mechanism. Since this method combines the advantages of self-attention mechanism and LSTM, the accuracy and performance of emotion recognition will be improved. The main work of this article is listed as follows:

(1) An improved LSTM model is used for speech feature extraction. The improved LSTM adds a peephole connection. In the original LSTM structure, the previous unit state c<t-1> is connected to the forget gate and input gate, and the state of the previous unit is added to the forget gate and input gate. Calculating. The current state will not lose the information obtained by the upper state, and the output at the next moment will be more specific and complete.
(2) A self-attention mechanism is introduced. This mechanism is a strategy calculated according to the importance of different parts of things. The goal is to allocate more attention to the key parts of things. Through the calculation of the attention probability distribution, a larger weight is assigned to a certain key part. After the speech features are output by LSTM, the weight value of each frame is calculated. The introduction of this mechanism allows information with a large contribution rate to be assigned a high weight.
(3) Based on the self-attention mechanism and ILSTM, a speech-based emotion recognition method is proposed. Compared with the traditional LSTM, the method used in this paper has improved the accuracy of emotion recognition on multiple data sets.
(4) Based on the emotion recognition function, an emotional interaction design plan is proposed. The advantage of emotional interaction design proposed in this research lies in more accurate emotion recognition and different interaction designs based on the recognition results. The ultimate goal of this research is to enhance the user's product experience and truly put people first.

## Related Information

### Emotional Design Concept

People's happiness, anger, worry, thought, fear, and shock are all emotions. Emotions refer to the feelings produced by the interaction between life phenomena and the human heart. Emotional design is to give and satisfy people's emotional needs for a product. By fully considering the needs of human nature, the product that had no vitality was added to the emotional element to realize the spiritual function of the product. In 2002, the “emotional” concept of product design was proposed by American cognitive psychologist Donald Norman. In 2004, the book “Emotional Design” was published, which pushed the research of emotionalization from behind the scenes to the front, and attracted widespread attention. Nowadays, the Internet environment is becoming more mature, and market competition is increasingly encouraging. Products that simply solve functional requirements can no longer meet the needs of users. People's needs are developing toward higher levels such as emotion and interaction. This requires the combination of emotional design theory and interaction design, and through emotional interaction to meet the increasingly individual and diverse needs of users.

### Principles of Emotional Design

#### Principles of Emotional Expression

The interactive design of the product needs to satisfy the user's emotional expression, as much as possible to satisfy the user's desire for expression, and cannot ignore the user's emotional appeal. For example, when using various social software, users can set an avatar, cover or change the theme. This is to satisfy the user's desire for expression as much as possible while transmitting information.

#### The Principle of Fun

The expression of the design should be humanized, as far as possible to draw the distance between man and machine. For example, in the process feedback, the two modes of “loading” and “loading hard” are displayed, the latter is more likely to give users a good impression. For the 404 page prompt, the non-emotional design is to only display “the requested page does not exist or the connection is wrong.” The emotional design is to display “the page you were looking for ran away from home,” coupled with a anthropomorphic picture full of sorry. As a user, the latter is more likely to forgive or understand emotions. Emotional design links the functions of the product with the emotions between users, which greatly increases the user's acceptance.

### Interaction Design Framework Based on Emotion Recognition

**Figure 2:**
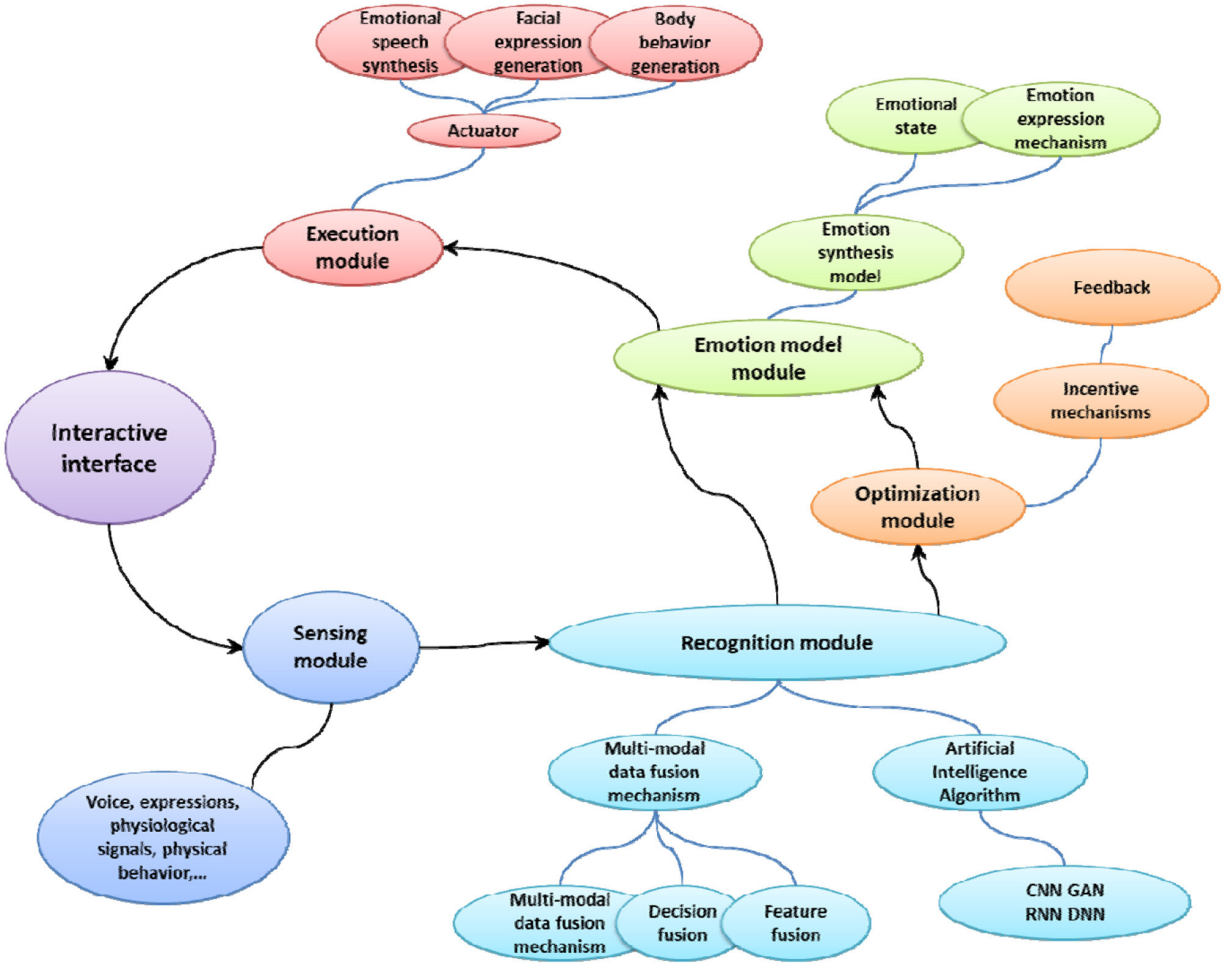
Interaction design framework based on emotion recognition.

The goal of emotional interaction is to make the computer make reasonable adjustments under the premise of understanding the human emotional state, and adapt to the transfer of user emotions. The focus of emotional interaction design is to use a variety of perception methods to recognize, interpret and respond to human emotions. Figure 2 shows the framework of interaction design based on emotion recognition. The interactive design system mainly includes a sensing module, an execution module, a recognition module, an emotion calculation module, and an optimization module.

The sensor module is the basis of the emotional interaction system. It uses a variety of sensor devices such as microphones, cameras, wearable devices and eye trackers to collect biometric data, including electrical signals in the brain, ECG signals, electrothermal signals, speech signals, facial expressions and physical behaviors, etc. The sensor module is equivalent to the neuron on the human body, feeling the stimulation of external substances all the time.

The recognition module is the basis of emotional interaction. First, it is necessary to preprocess the collected data. Then, the features of the data sample are extracted. Finally, the feature data is classified to obtain the recognition result. Relevant studies have shown that the use of deep learning methods, such as recurrent neural networks (RNN), convolutional neural networks (CNN), and adversarial neural networks (GAN), have higher speculation potential. They can not only automatically generate the best method for detecting the subjective experience of the user's emotion, but also model the user's long-term behavior through the storage device. In addition, emotion recognition based on multi-modal user information can choose different modal combinations, and there are multiple choices for the data fusion method between modalities according to actual conditions.

The emotion model module is a key part of emotion recognition and emotion expression. By establishing a mathematical model of emotional state, a more reasonable emotional understanding and feedback can be achieved. The emotional model module needs to study various psychological phenomena of the user, and make a reasonable response to the user's emotions by judging the composition of the emotional information. The expression of emotion, as a branch of experience and classic theoretical research, needs to build a human emotion model with inherent utility, summarize cognition from reality, and verify it through experiments.

The execution module is responsible for the information output task of the emotional interaction system. Perform corresponding feedback according to the decision made by the emotion model module, and finally complete the emotional interaction through emotional expression such as facial expressions, body movements, and synthesized speech.

In the emotional interaction system, the optimization module is the key link to provide personalized services. Since it is impossible to find an emotional model solution suitable for all systems, emotional modeling is still highly dependent on user feedback information, so it is particularly important to have an adaptive optimization module. The optimization plan is usually completed in a controlled interactive environment, and the user's response to a specific product needs to be monitored. Monitoring information can provide valuable feedback information for the system, so as to establish a good and personalized reward and punishment optimization mechanism to achieve a better user experience.

## Emotion Recognition Based on LSTM

**Figure 3:**
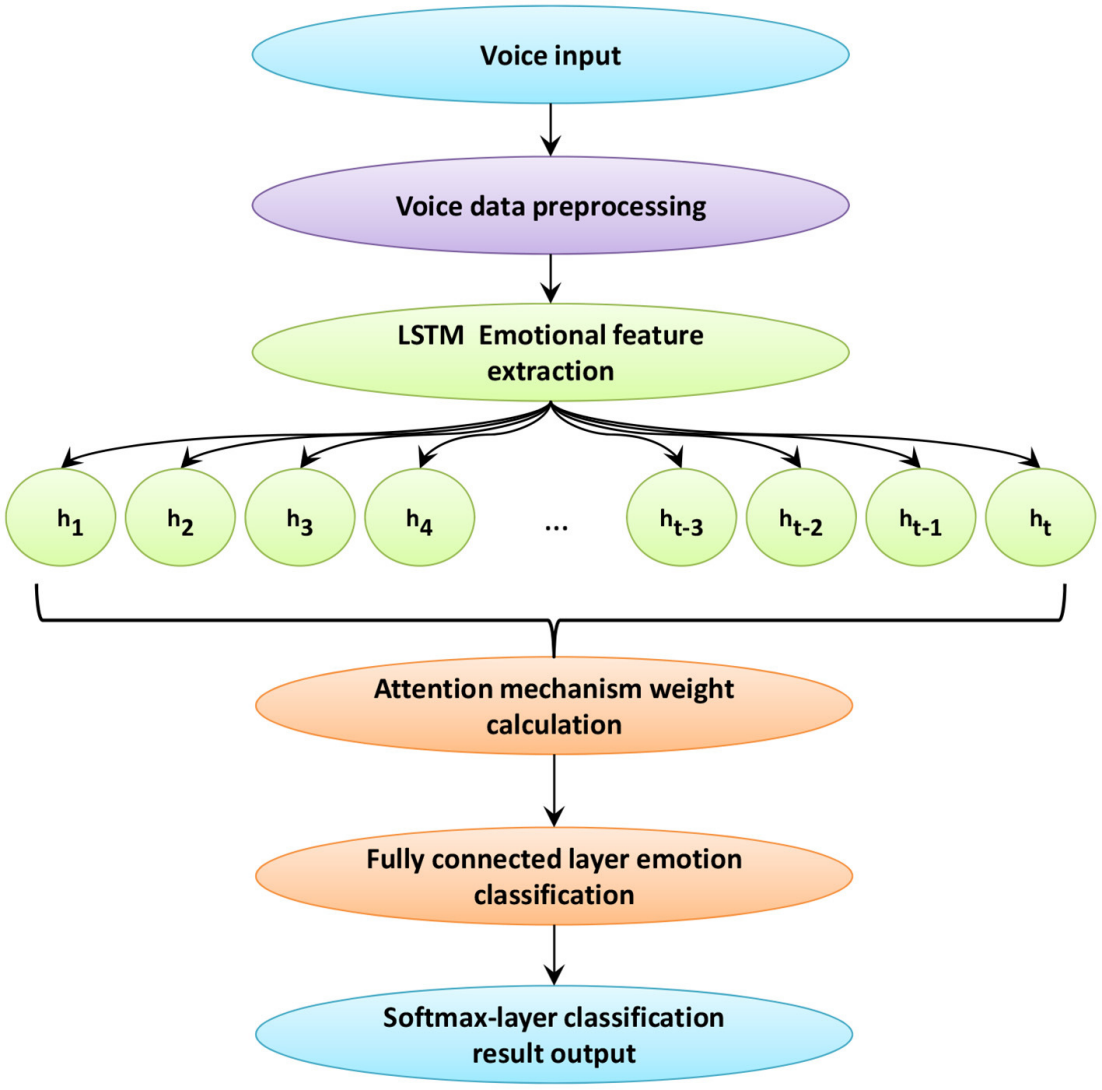
Speech recognition framework based on the method in this paper.

This article uses a speech-based emotion recognition framework, the framework diagram is shown in Figure 3. *h*_*i*_, *i* = 1, 2, …, *t* is the speech sequence of each frame obtained after passing through LSTM. *t* is the number of frames of the speech signal. In this framework, the input speech signal is preprocessed first. Then, perform MFCC extraction on the speech signal through the opensmile tool. The features extracted by MFCC are input into the ILSTM model. By adding ILSTM with peephole connection, a complete speech sequence is obtained. Then, the matrix output in ILSTM is used as the input of the attention mechanism layer. In the attention mechanism layer, through similarity calculation, the attention weight of each frame of speech signal relative to the recognition target is learned. Multiply the learned weight value with the input matrix to get the final weight value. Finally, the obtained information is classified by the fully connected layer, and the output of the speech emotion recognition result is realized by the Softmax layer.

### MFCC Extraction

Traditional speech features are mainly divided into acoustic features, prosodic features and sound quality features. Due to the complexity of speech emotions, many emotions are difficult to effectively recognize based on prosody and sound quality alone, which results in a single original feature that is not well distinguished. This requires different speech features to be combined for speech recognition. Moreover, the non-stationary random process of speech features has a strong time-varying nature. Therefore, in order to increase the practicability of feature parameters and reduce the complexity of feature extraction, MFCC is selected as the speech emotion feature in this research. The calculation relationship between MFCC and human ear frequency can be expressed by Eq. (1):

(1) $$\begin{array}{r} M\left(f\right)=2595\times \log_{10}\left(1+\frac{f}{700}\right) \end{array}$$

**Figure 4:**
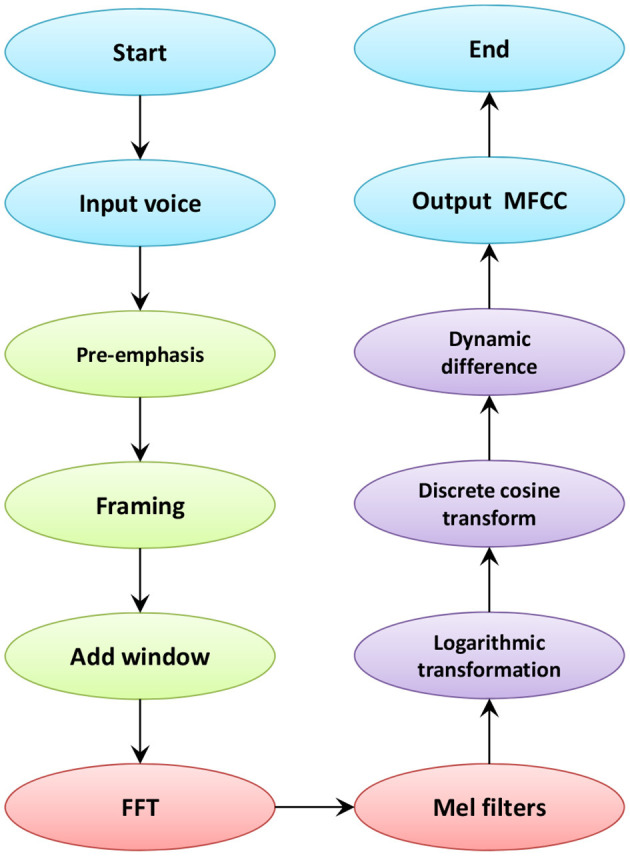
The process of MFCC extraction.

Where *M* represents the Mel frequency function and *f* represents the linear frequency. Eq. (1) shows the relationship between Mel frequency and linear frequency. Within the Mel frequency, the human perception of audio is linear. MFCC coefficient is to construct characteristic parameters by simulating human ear characteristics and human hearing characteristics. The process of MFCC extraction is shown in Figure 4.

As shown in Figure 4, the speech signal first passes through the first-order high-pass filter of the transfer function for pre-emphasis. This kind of preprocessing can enhance the high-frequency components of the signal and compensate for the lost speech signal. Then, in order to ensure the short-term stability of the speech signal, it is necessary to perform frame processing. The speech signal is divided into several segments, and each short segment is called a frame, which improves the continuity between the left and right ends of the frame. After that, windowing is performed. The traditional MFCC coefficient windowing operation is to add a Hamming window. In this paper, the ordinary Hamming window is changed to a fourth-order new Hamming window. Eq. (2) is the calculation formula that adds the second harmonic component and fourth harmonic component of cosine.

(2) $$\begin{array}{r} w\left(n\right)=\left(a+b\cos\frac{2\pi n}{N-1}+c\cos\frac{4\pi n}{N-1}+d\cos\frac{8\pi n}{N-1}\right)R_{N}\left(n\right) \end{array}$$

Where a, b, c, d are the orientation coefficients of the window function, *N* is the window length, and *R*_*N*_ is the time domain sequence of the rectangular window of the current window length. The data in the windowed speech undergoes Fast Fourier transform frame by frame. After taking the square of the absolute value, the frequency spectrum of each frame of speech signal can be obtained. Perform modulo operation and square operation on the acquired spectrum to generate the power spectrum of the speech signal. The energy spectrum is passed through a set of Mel-scale triangular filters to eliminate the effect of harmonics. Usually a filter bank contains 22–26 filters. The number of Mel filters used in this article is 128. After calculating the logarithmic energy output by each filter bank, the calculation result is brought into the discrete cosine transform. Most of the signal data is concentrated in the low frequency area. To eliminate redundant data, the first 25 data are taken. Then, the logarithmic energy of one frame is added to extract the dynamic difference parameters to calculate the signal difference of different frames. Finally, the global speech signal cepstrum parameters are used as static features, and the difference spectrum of static features is used as dynamic features. The calculation method of differential parameters combines static characteristics and dynamic characteristics to improve the recognition performance of the system.

### Feature Extraction Based on ILSTM

**Figure 5:**
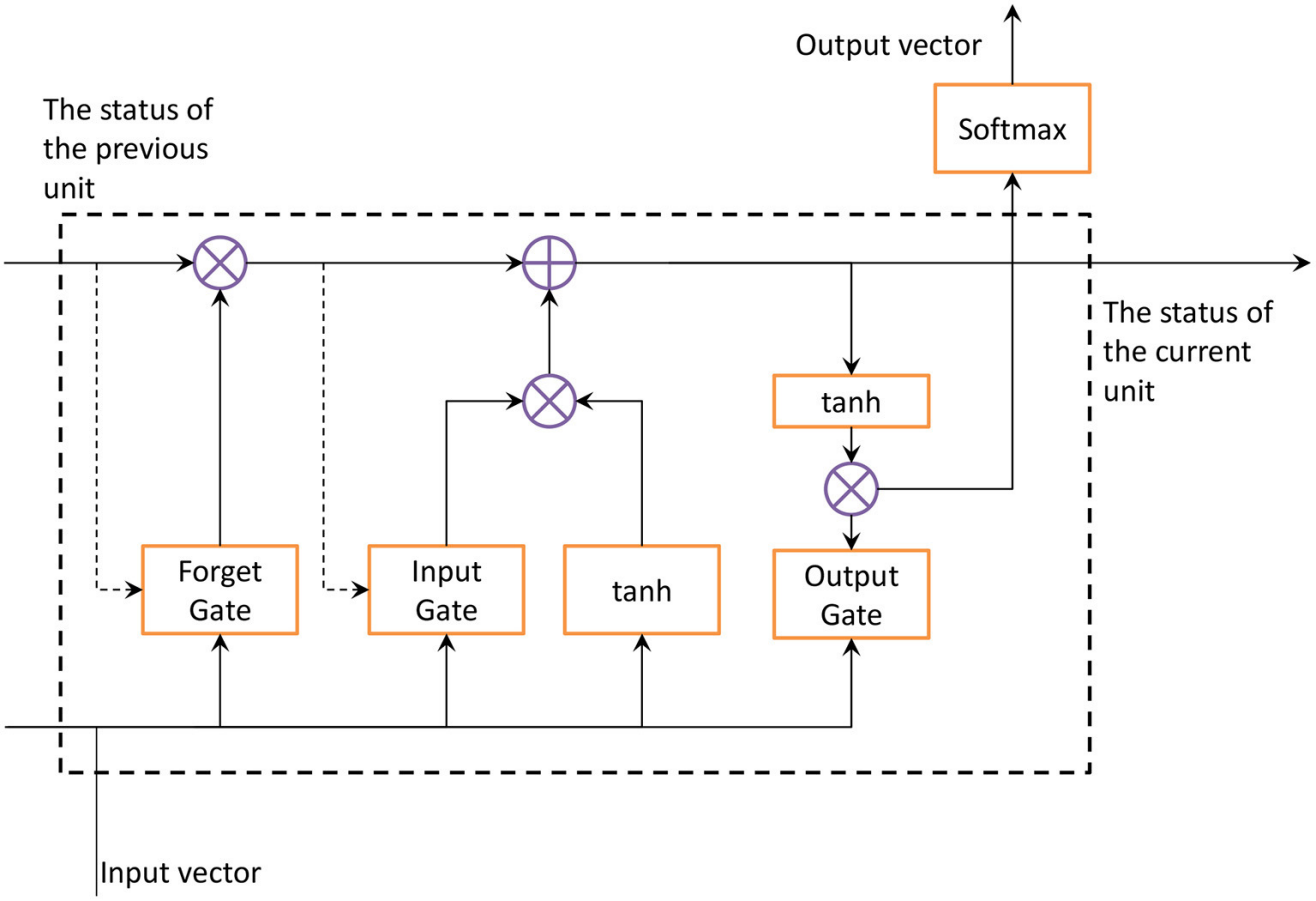
ILSTM structure diagram.

The feed forward neural network of the LSTM network accepts the input of a specific structure, and the LSTM cyclically transmits the state in its own network. Therefore, the input range of time series structure type data is wider, and it has the function of describing dynamic time behavior. The key to LSTM is the unit state. The transfer effect of the unit state exists in the entire chain structure. There is a small amount of linear interaction in the process of transmission, so that information is easy to get. LSTM removes or adds information to the cell state through three gates. A gate is a structure that allows information to pass through selectively. An LSTM unit is composed of forget gate, input gate and output gate. Multiple units in the LSTM network form a series structure. LSTM was created to avoid the dependence of RNN from being affected by the long-term previous state. In the three gating units, the calculation of each unit will lose the information processed in the previous sequence. To ensure the integrity of the information and the accuracy of the output at the next moment, this study uses the ILSTM algorithm for speech emotion recognition. ILSTM adds peephole connections to the traditional LSTM network, and the model structure is shown in Figure 5. The dotted line in Figure 5 is the added peephole connection. The function of this connection is to connect the last unit state c<t-1> in the traditional LSTM structure with the forget gate and the input gate. By adding the state of the previous unit to the calculation of the forget gate and input gate, the current state will not lose the information already obtained by the upper state, making the output at the next moment more complete.

The function of the forget gate is to select the information to be discarded from the cell state. The gate reads the output vector and the input of the memory unit, and outputs a value in the interval (0, 1) through a sigmod function. The values at both ends of the interval represent completely discarded or completely reserved. ILSTM adds the state of the previous unit to the sigmod function. The state of the previous unit is taken into account in the calculation of the forgetting gate, which makes the selection of the forgetting layer more accurate and prevents part of the effective information from being forgotten. The calculation expression of the forget gate at time *t* is:

(3) $$\begin{array}{r} F_{t}=\sigma \left({\text{W}}_{F}\left[c^{\langle t-1\rangle },\alpha^{\langle t-1\rangle },{\text{x}}^{\langle t\rangle }\right]+{\text{b}}_{F}\right) \end{array}$$

where *F*_*t*_ represents the forgetting gate at time *t, c, a*, and *x* represent the state of the memory unit, the output vector and input vector of the memory unit, respectively.**W**_*F*_ and **b**_*F*_ represent the weight matrix and bias vector in the forgetting gate unit. The input gate is calculated according to the last output and current input value, and the addition of new state information is controlled by the degree of update. The unit state of the upper layer is also added to the input gate. The information added to the unit state must be important so that the update effect can be more accurate. The update formula of the input gate and current state at time t is:

(4) $$\begin{array}{r} I_{t}=\sigma \left(W_{I}\left[c^{\langle t-1\rangle },\alpha^{\langle t-1\rangle },{\text{x}}^{\langle t\rangle }\right]+{\text{b}}_{I}\right) \end{array}$$

(5) $$\begin{array}{r} c^{\langle t\rangle }=I_{t}\times {\bar{c}}^{\langle t\rangle }+F_{t}\times {\bar{c}}^{\langle t-1\rangle } \end{array}$$

(6) $$\begin{array}{r} {\bar{c}}^{\langle t\rangle }\text{=}\tanh\left({\text{W}}_{c}\left[\alpha^{\langle t-1\rangle },{\text{x}}^{\langle t\rangle }\right]+{\text{b}}_{c}\right) \end{array}$$

where *I*_*t*_ represents the input gate at time *t*, and *W*_*I*_ and **b**_*I*_ represent the weight matrix and bias vector in the input gate unit. After adding the previous unit state to the forget gate and input gate, the current unit state will be more accurate as the two gates are improved. The forget gate and the input gate and the update unit simultaneously control the current unit status, thereby improving the accuracy of the current unit status. Finally, the output result obtained by the unit state dot multiplication output gate is more comprehensive. To reduce complexity, no peephole connection is added to the output gate. Then the formula of the output gate at time *t* is:

(7) $$\begin{array}{r} {\text{O}}_{t}\text{=}\sigma \left({\text{W}}_{o}\left[\alpha^{\langle t-1\rangle },{\text{x}}^{\langle t\rangle }\right]+{\text{b}}_{o}\right) \end{array}$$

where O_*t*_ represents the output gate at time *t*, and **W**_*o*_ and **b**_*o*_ represent the weight matrix and bias vector in the output gate unit. The output gate controls the effect of long-term memory on the current output. The final output of the network is determined by the input gate, forget gate, output gate, the state of the previous memory unit and the state of the current memory unit.

### Self-Attention Mechanism

**Figure 6:**
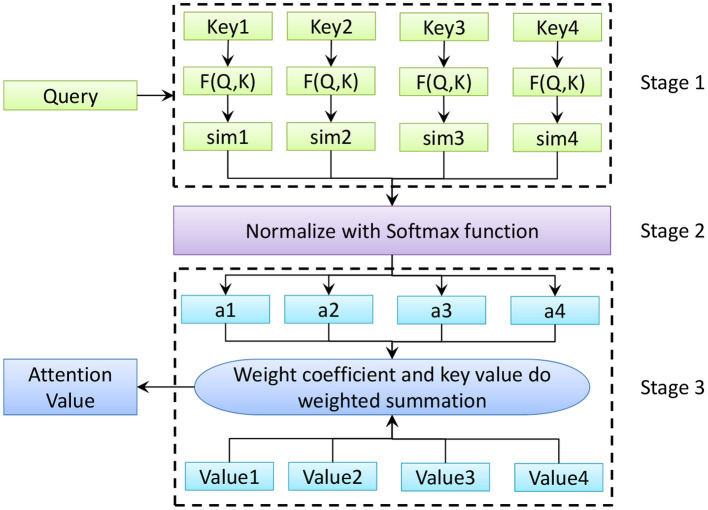
Self-attention mechanism calculation process.

The essence of the self-attention mechanism is a mapping relationship between keys and values. Key-value query contains three basic elements: query, key, and value. The weight coefficient of each key value is obtained by calculating the correlation between each query item and each key. Then the weight and the corresponding key value are weighted and summed. After the speech signal is input to ILSTM, the weight value of each frame is calculated. The calculation process of the self-attention mechanism is shown in Figure 6. In Figure 6, Key, Query, and Value, respectively represent the key word of the input feature, the query value and the weight value of the current key word. *F* represents the function for calculating the weight coefficient. *Sim* represents the similarity obtained by the weight coefficient. *a* represents the weight coefficient of the corresponding key value.

As shown in Figure 6, the self-attention mechanism can be divided into three stages. In the first stage, the weight coefficient of each keyword's corresponding value is obtained by calculating the correlation between each query value and each keyword. There are generally three calculation methods, namely the vector dot product method, the cosine function method and the introduction of additional neural network evaluation methods. The three calculation formulas are as follows

(8) $$\begin{array}{r} S\text{im}ilarity\left(Query,Keyi\right)=Query□Keyi \end{array}$$

(9) $$\begin{array}{r} S\text{im}ilarity\left(Query,Keyi\right)=\frac{Query□Keyi}{\left||Query||□||Keyi|\right|} \end{array}$$

(10) $$\begin{array}{r} S\text{im}ilarity\left(Query,Keyi\right)=MLP\left(Query,Keyi\right) \end{array}$$

In the second stage, the Softmax function is used to normalize the weights. Eq. (11) is the normalized processing formula. *L*_*x*_ represents the length of the corresponding data source.

(11) $$\begin{array}{r} \alpha_{i}=soft\max\left(Sim_{i}\right)=\frac{e^{Sim_{i}}}{\overset{L_{x}}{\underset{j=1}{\sum }}e^{Sim_{j}}} \end{array}$$

In the third stage, the weight coefficient and the corresponding key value are weighted and summed to obtain the final attention value, as shown in Eq. (12).

(12) $$\begin{array}{r} Attention\left(Query,Source\right)=\overset{L_{x}}{\underset{i=1}{\sum }}\alpha_{i}\cdot Value_{i} \end{array}$$

Suppose there are T frames after the speech data is divided, and the latitude of each frame is the number of neurons in the ILSTM network. So the size of the matrix obtained after passing through the ILSTM network is n^*^T, where n is the dimension of each frame of speech. The encoding output of the attention layer is obtained through the calculation of Eq. (13).

(13) $$\begin{array}{r} A=soft\max\left(g\left({\text{H}}^{T}{\text{W}}_{1}\right){\text{W}}_{2}\right) \end{array}$$

**W**_1_ and **W**_2_ represent the most suitable parameter matrix given manually. **H** represents the input matrix extracted by ILSTM. Finally, the weight value calculated by the self-attention mechanism is multiplied by the input matrix **H**, and the result of the multiplication is input to the fully connected layer for classification.

## Experimental Process and Result Analysis

### Experimental Data Set

Two experimental data sets are used in this experiment. The basic situation of each data set is shown in Table 1. During the experiment, 30% of the entire data set was selected as the test data set and 70% as the training data set.

**Table 1:** Data set introduction.

**Database**	**Number of categories**	**Category breakdown**	**Number of samples**	**Sampling rate**
EMO-DB	7	Neutral, angry, scared, happy, sad, disgusted, bored	535	48 kHz
CASIA	6	Happy, sad, scared, angry, surprised, neutral	9,600	16 kHz

### Experimental Setup

The comparison methods used in the experiment are LSTM, ILSTM, and the method used in this article. The evaluation index is accuracy. The main hardware configuration of the experimental platform used in this article is: the processor is Intel Xeon processor E5-2600 @ 3. 40 GHz, the GPU is NVIDIA GeForce GTX1080Ti, and the memory is 64G. Other experimental parameter settings are shown in Table 2.

**Table 2:** Experimental parameter settings.

**Parameter**	**Setting instructions**
Number of Mel filters	128
Number of hidden layer units of LSTM/ILSTM	128
Learning rate	0.01
Speech signal	16 bit

### Discussion of Experimental Results

#### Emo-DB

To compare the actual recognition effect of each method on the Emo-DB database, this section applies each method to the Emo-DB data set. The experimental results are shown in Tables 3–5. Comparing the data in the three tables, it can be seen that the recognition rates of the four emotions all exceed 60%. These four emotions are anger, boredom, disgust, and sadness. The recognition rate of the remaining three emotions is <50%. Natural emotions are easily confused with boring emotions, and angry emotions are easily confused with fear emotions. The recognition rate based on the traditional LSTM model is 62.35%, the recognition rate based on the ILSTM model is 63.57%, and the recognition rate based on this method is 65.29%. Compared with traditional LSTM, the recognition rate of ILSTM has increased by nearly 3%. The recognition rate of this method has increased by nearly five percentage points. This fully demonstrates the effectiveness and superiority of this method.

**Table 3:** Recognition rate of traditional LSTM in Emo-DB.

**Emotion category**	**Angry**	**Bored**	**Disgust**	**Fear**	**Happy**	**Natural**	**Sad**
Angry	**75**	2	3	3	15	1	1
Bored	0.5	**88**	0	0	2	5	4.5
Disgust	15	11	**67**	1.5	0.5	5	0.0
Fear	21	14	5	**32**	19.5	3.5	5
Happy	54.5	0	3	1	**42**	0	0.5
Natural	1	57	0	0.5	0.5	**39**	2.0
Sad	1.5	5	0	0	0	0	**93.5**

*The bold values represent the recognition rate of the adopted method*.

**Table 4:** Recognition rate of ILSTM in Emo-DB.

**Emotion category**	**Angry**	**Bored**	**Disgust**	**Fear**	**Happy**	**Natural**	**Sad**
Angry	**74**	3	3	2.5	17	0	0.5
Bored	0.5	**91**	0	0	1	4	3.5
Disgust	13	11	**70.5**	1	0	4.5	0.0
Fear	20	16	5	**29.5**	19	4	5.5
Happy	50	0	3	1	**45**	0	1
Natural	1	49	0	0.5	0.5	**44**	5.0
Sad	1	7	1	0	0	0	**91**

*The bold values represent the recognition rate of the adopted method*.

**Table 5:** Recognition rate of this method in Emo-DB.

**Emotion category**	**Angry**	**Bored**	**Disgust**	**Fear**	**Happy**	**Natural**	**Sad**
Angry	**77**	2	3	2	16	0	0
Bored	0	**93**	0	0	1	4	2
Disgust	13	10	**71**	1	0	5	0.0
Fear	20	16	4	**31**	19	5	5
Happy	49	1	3	1	**46**	0	0
Natural	1	48	0	0	0	**46**	5.0
Sad	0.5	5.5	1	0	0	0	**93**

*The bold values represent the recognition rate of the adopted method*.

Comparing the recognition of various emotions by traditional LSTM and ILSTM, the recognition rates for the five emotions of boredom, disgust, fear, happiness, and nature have all increased by more than 2.5%. The recognition rate of anger and fear emotions has decreased slightly. But on the whole, ILSTM's recognition rate of emotion has improved. This shows that because ILSTM adds peephole connections in the input gate and the forget gate, the information reception is more complete and the state extraction is more sufficient. In the calculation of each unit layer, the unit state of the upper layer is input into the input gate and the forget gate, which makes the speech signal reception more complete, thereby improving the overall recognition performance.

Comparing the recognition of various emotions by traditional LSTM and the method in this paper, the recognition rates of the five emotions of boredom, disgust, fear, happiness, and nature have all increased by more than 4%. The recognition rate of angry emotions increased by 2%. The recognition rate of sad emotions decreased slightly. It can be seen from the results that the method used in this paper has the best recognition rate. This is because ILSTM is sufficient for information extraction. The speech data is divided into frames and then input into the attention mechanism. The importance of each frame of signal is learned in the attention layer, and the output is weighted. Because the output information of ILSTM is more specific, the weight of each frame of speech is more accurate.

#### CASIA

The experimental results of each method on the CASIA data set are shown in Tables 6–8. Comparing the data in the three tables, it can be seen that the recognition rates of the four emotions all exceed 60%. These four emotions are anger, happiness, calm, and surprise. The recognition rate of the remaining two emotions is close to 50%. The data in the table shows that it is easy to confuse fear and sadness. The recognition rate based on traditional LSTM is 68.75%, the recognition rate based on ILSTM is 70.75%, and the recognition rate based on this method is 74.17%. Compared with traditional LSTM, the recognition rate of ILSTM is increased by 2%. The recognition rate of this method has increased by five percentage points. This fully demonstrates the effectiveness and superiority of this method.

**Table 6:** Recognition rate of traditional LSTM in CASIA.

**Emotion category**	**Angry**	**Happy**	**Fear**	**Calm**	**Surprised**	**Sad**
Angry	**77**	11	2	1	7.5	1.5
Happy	8	**69.5**	5	3.5	13	1
Fear	2	6	**53**	2	4	33
Calm	1.5	7.5	10	**77**	2	2
Surprised	8	7	2	3	**79**	1
Sad	3	1.5	33	3.5	2	**57**

*The bold values represent the recognition rate of the adopted method*.

**Table 7:** Recognition rate of ILSTM in CASIA.

**Emotion category**	**Angry**	**Happy**	**Fear**	**Calm**	**Surprised**	**Sad**
Angry	**77.5**	10.5	1	1	8	2
Happy	5	**75**	2	3	14	1
Fear	3	5	**57**	1	2.5	31.5
Calm	2	8	9.5	**75**	1.5	4
Surprised	7	6	3	2	**81.5**	0.5
Sad	2	3	34	2.5	0	**58.5**

*The bold values represent the recognition rate of the adopted method*.

**Table 8:** Recognition rate of this method in CASIA.

**Emotion category**	**Angry**	**Happy**	**Fear**	**Calm**	**Surprised**	**Sad**
Angry	**78**	9	3	0	9	1
Happy	4	**78**	5	2	11	0
Fear	1.5	2.5	**59**	1	5	31
Calm	1	4	10	**77**	6.5	1.5
Surprised	6	5	4	1	**83**	1
Sad	2	2	30.5	1.5	3	**60**

*The bold values represent the recognition rate of the adopted method*.

Comparing the recognition of various emotions by traditional LSTM and ILSTM, the recognition rate of the five emotions of anger, happiness, fear, surprise, and sadness has increased, with an average increase of 2.8%. The recognition rate of calm emotion dropped by 2%. But on the whole, ILSTM's recognition rate of emotion has improved. Comparing the recognition of various emotions by traditional LSTM and the method in this paper, the recognition rate of the five emotions of angry, happy, scared, surprised and sad has increased, with an average increase of 4.5%. The recognition rate of calm emotion has not changed. On the whole, ILSTM's recognition rate of emotion has improved. Comparing the recognition results of the three methods, it can be seen that the recognition rate of this method is the best.

## Conclusion

The key to emotional interaction design is accurate emotional recognition results. Based on this requirement, this article uses an emotion recognition framework based on LSTM. The framework first extracts the MFCC features of the speech information. Second, ILSTM performs feature extraction on MFCC. The output of the entire ILSTM is used as the input of the attention layer, which is input to the fully connected layer and the Softmax layer after weight calculation to obtain the final recognition result. The recognition results on Emo-DB and CASIA data sets prove that the method in this paper has the best recognition effect. With the development of emotion science, computer science, and electronics, the widespread application of intelligent algorithms and wearable technology has made emotional interaction possible. These new technologies have become part of people's real lives and help to continuously improve the quality of life. In addition, these technologies can obtain large amounts of data, which can be used to develop complex artificial intelligence algorithms to achieve reliable emotional algorithm calculation models. Many methods have been developed for emotional design, and new research questions, challenges and opportunities have emerged, which have made people's understanding and cognition of emotional design a step forward.

In the next step, the method of this paper will be introduced into the fields of machine translation and polygraph detection, to test the continuous emotion corpus and improve the calculation of attention scores to further improve the speech emotion recognition rate.

## Data Availability Statement

The original contributions presented in the study are included in the article/supplementary material, further inquiries can be directed to the corresponding author/s.

## Author Contributions

XC and LZ conceived and developed the theoretical framework of the manuscript. XC, RH, and XL carried out model design, theoretical verification, and training under the guidance of RH. XC, LX, and MZ conducted the evaluation and analysis of experimental data under the guidance of RH. Everyone participated in the process of writing and revising the manuscript.

## Conflict of Interest

The authors declare that the research was conducted in the absence of any commercial or financial relationships that could be construed as a potential conflict of interest.
